# OsABCG9 Is an Important ABC Transporter of Cuticular Wax Deposition in Rice

**DOI:** 10.3389/fpls.2018.00960

**Published:** 2018-08-07

**Authors:** Van N.T. Nguyen, Saet Buyl Lee, Mi Chung Suh, Gynheung An, Ki-Hong Jung

**Affiliations:** ^1^Graduate School of Biotechnology and Crop Biotech Institute, Kyung Hee University, Seoul, South Korea; ^2^Department of Bioenergy Science and Technology, College of Agriculture and Life Sciences, Chonnam National University, Gwangju, South Korea

**Keywords:** ABC transporter, ABCG9, wax transportation, cutin transportation, rice

## Abstract

The importance of the cuticular layer in regulating a plant’s water status and providing protection from environmental challenges has been recognized for a long time. The cuticular layer in plants restricts non-stomatal water loss and protects plants against damage from pathogen infection and UV radiation. Much genetic and biochemical research has been done about cutin and wax transportation in *Arabidopsis thaliana*, but little is known about it in rice. Here, we report that a rice ATP-binding cassette (ABC) transporter, *OsABCG9*, is essential for normal development during vegetative growth and could play a critical role in the transportation of epicuticular wax in rice. Rice phenotypes with mutated *OsABCG9* exhibited growth retardation and sensitivity to low humidity. The total amount of cuticular wax on the leaves of the *osabcg9-1* mutant diminished by 53% compared with the wild type, and wax crystals disappeared completely in *osabcg9-2* mutant leaves. However, OsABCG9 does not seem to be involved in cutin transportation, even though its ortholog in *Arabidopsis*, AtABCG11, transports both wax and cutin. Furthermore, the *osabcg9-1* mutant had increased leaf chlorophyll leaching and more severe drought susceptibility. This study provides new insights about differences between rice and *A. thaliana* in wax and cutin transportation associated with the ABCG family during evolution.

## Introduction

To protect themselves from biotic and abiotic stress in the external environment, the outermost layer of the aerial organs of terrestrial plants are covered with a hydrophobic layer called the cuticle or cuticular membrane (Heredia, 2003). Though the structure and components vary among plant species, the cuticle is generally composed of two highly lipophilic layers: a wax layer on the outer surface of a cutin layer (Heredia, 2003; Hwang et al., 2016). Cutin, the backbone of the cuticle, is a polymer including mainly ω- and mid-chain hydroxy and epoxy C_16_ and C_18_ fatty acids esterified into glycerol (Heredia, 2003; Hwang et al., 2016). In contrast, cuticular wax is monomeric and predominantly consists of very long chain fatty acids and their derivatives, including secondary metabolites such as triterpenoids, phenylpropanoids, and flavonoids (Kunst and Samuels, 2003; Jetter et al., 2007).

Cuticular wax and cutin are exported from epidermal cells to the cuticle, where they play essential roles in non-stomatal transpiration (Riederer and Schreiber, 2001) and protection against pathogenic microbes and herbivorous insects (Eigenbrode and Espelie, 1995) and UV exposure (Krauss et al., 1997). Because of those important protective functions, researchers are increasingly interested in the genes related to the cutin and wax pathways. Wax compounds are biosynthesized from very long chain fatty acid precursors, and cutin forms from C16–C18 fatty acids and their derivatives. The production of both substances occurs mostly in the endoplasmic reticulum (ER) of the epidermal cells (Samuels et al., 2008). Wax and cutin are transported to the plasma membrane and need a transporter to get through the plasma membrane.

ATP-binding cassette transporter (ABC-transporter) is one of largest protein family plays role in transportation of various substrates through membranes, wax and cutin are also among them. In *Arabidopsis, CER5* (*AtABCG12*) was the first plasma membrane transporter, that define as ABC transporter, related to cuticular lipids transportation to be found (Pighin et al., 2004). A *cer5* mutant had 41% of the wild-type wax load only in the stem, even though *CER5*/*AtABCG12* is also expressed in other plant organs, such as the leaves, siliques, flowers, and roots. That suggests the extra transporters are involved in wax secretion. *AtABCG11* gene was identified latterly as the gene shares a high expression correlation with *CER5*/*AtABCG12* by Genevestigator (Bird et al., 2007). Surprisingly, AtABCG11 is involved not only in cuticular wax but also in cutin monomer transportation (Bird et al., 2007; Luo et al., 2007; Panikashvili et al., 2007), and it forms a homodimer or a heterodimer with CER5/AtABCG12. And the function of CER5/AtABCG12 protein depends on its interaction with ABCG11 (McFarlane et al., 2010). *AtABCG13* also participates in the secretion of cuticle monomers in flowers, particularly petals (Sánchez-Fernández et al., 2001). Another ABC transporter studied in *Arabidopsis* is *AtABCG32*, which exports cuticle precursors in petals (Bessire et al., 2011)

In rice, ABC transporter family composes 126 genes were classified into eight subgroups base on their protein size, orientation and transmembrane domain (Nguyen et al., 2014). In particular, the ABCG subgroup has a highest number of members, up to 50 genes and most of them are still functionally unidentified yet. Recently, *OsABCG5* was characterized as having a role in the suberization of the hypodermis of rice roots (Shiono et al., 2014). Mutant of *OsABCG15* affects to the structure of pollen exine and anther cuticle (Wu et al., 2014). While the biological functions of cuticular lipid transporters in leaf have been intensively studies in *Arabidopsis thaliana*, those in rice remain very limited. Addition, the diversity of wax and cutin structures and compositions across various land plants in many reports have been described (Macey and Barber, 1970; Jenks et al., 1995; Barthlott et al., 1996; Meusel et al., 1999; Markstädter et al., 2000). For this study, we investigated the function of OsABCG9, which has 78% similarity to AtABCG11, by using T-DNA insertional lines. We found that, unlike AtABCG11, OsABCG9 plays an important function relating wax composition in rice.

## Materials and Methods

### Plant Material, Growth Conditions, and Stress Treatments

Rice (*Oryza sativa* cv. Dongjin) lines used in this study were isolated from a T-DNA insertional mutant population (POSTCH population in^1^ Jeon et al., 2000): lines 1B-03134 and 1B-22818. Sterilized seed were germinated on one-half-strength Murashige and Skoog solidified medium for 1 week under continuous light at 28°C. The plants were then transferred to soil for further development under greenhouse or paddy field conditions.

To determine the phenotypes of the *osabcg9* mutants in response to water deficiency stress at the young seedling stage, wild type plants and two mutant alleles were exposed in air for up to 1 h. For 5-week-old seedlings, water was withdrawn from pots growing wild type and mutant plants, and the seedlings were exposed to drought stress for 3 days (Yoo et al., 2017). Then, we added water to the container and grew the plants for 7 days so they could recover from the drought stress.

### Quantitative Real-Time PCR Assays

Total RNA was obtained using Trizol following the manufacturer’s protocols and synthesized to complementary DNA (cDNA) by reverse transcriptase (Promega Corp., Madison, WI, United States). The expression of genes was estimated using Qiagen’s real-time PCR system with cycling conditions of 95°C for 30 s, 60°C for 30 s, and 72°C for 60 s. We used *rice ubiquitin 5* (*OsUbi5, LOC_Os01g22490*) as the internal control for all quantitative PCR reactions (Hong et al., 2017). The list of primers used in this study is given in **Supplementary Table S1**.

### Histochemical GUS Assay

To examine the expression of *OsABCG9*, the vector pGA3519, including a GUS reporter gene, was linearized by digestion using the XbaI restriction enzyme, and the 1921 bp region upstream of the annotated start codon of *OsABCG9* was amplified with a pair of primers with a 15-bp or longer extension homologous to each end of the linearized vector (**Supplementary Table S1**). Then, the fragment was introduced into a linear vector using a Takara In-Fusion Cloning Kit to create the *pOsABCG9::GUS* construct. The transformation was processed on hygromycin selection medium, and seeds in the T1 generation were used for the GUS analysis. Various tissues of the transgenic plants were incubated in a GUS solution overnight at 37°C after being vacuumed for 15 min, and then a 96% ethanol solution was exchanged at 65°C to remove chlorophyll. The stained tissues were monitored using an Olympus SZX16 microscope (Olympus, Tokyo, Japan). Semi-thin sections of rice stem were visualized using an Olympus BX61 microscope.

### Subcellular Localization

Transient expression of the OsABCG9-GFP fusion protein in onion epidermis cells was carried out and full-length cDNA of *OsABCG9* was amplified without a stop codon using primers containing the ECoRV and XbaI sites (**Supplementary Table S1**). Then, the fragment was inserted into a pGA3811 vector (*p35S::MSC::GFP*) cut by SmaI and SpeI restriction enzymes to make a GFP fusion vector. To observe OsABCG9 protein localization, we bombarded the vectors into onion epidermis via the biolistic particle delivery system. After 24 h of incubation under darkness at 28°C, the onion epidermal cells were examined under an Olympus BX61 microscope equipped with filter sets for GFP. The fusion vector *p35S::AtAHA2::RFP* was used as a plasma membrane marker (Yi et al., 2014).

Transient expression of the OsABCG9-GFP fusion protein in tobacco was carried out using *OsABCG9* cDNA cloned into pH7FWG2 vector (Karimi et al., 2002) via Gateway technology. The vectors were then transformed in *Agrobacterium tumefaciens* strain GV3101. *A. tumefaciens* cells were re-suspended in MMAi medium (5 g/L MS salts, 20 g/L sucrose, 10 mM MES and 200 μM acetosyringone) to OD_600_ 0.8 and then were infiltrated into *Nicotiana benthamiana* leaves. Infiltrated leaves were observed by confocal laser scanning microscopy (Zeiss LSM 510; Carl Zeiss, Jena, Germany) at three days after infiltration.

### Scanning Electron Microscopy Analysis

Ten-day-old leaves were fixed in 3% glutaldehyde-0.1 M sodium phosphate buffer (pH 7.2) at 4°C for 4 h. The samples were rinsed in a 0.1 M sodium phosphate buffer, pH 7.2, three times for 20 min and then dehydrated by a gradient ethanol series of 30 to 100% and infiltrated with an isoamyl acetate series. The samples were next placed under critical point dryer procession, platinum coated, and examined with a field emission scanning electron microscope (Zeiss Supra 55VP; Carl Zeiss, Jena, Germany).

### Transmission Electron Microscope Analysis

Samples were prefixed in 4% paraformaldehyde in a sodium phosphate buffer (pH 7.2), 0.1% TritonX-100, and 0.1% Tween 20 under vacuum for 1 h. We replaced the fixative with a fresh solution and rotated the samples overnight at 4°C. After washing in phosphate buffer, samples were post-fixed overnight in 2% (v/v) OsO_4_. After being dehydrated in an alcohol series, the samples were infiltrated with ethanol:Spurr mixtures in 1:1 and 1:3 ratios. Finally, the samples were infiltrated with pure Spurr resin and embedded into molds at 70–75°C for 12 h. Ultrathin cross-sections (80–100 nm) were prepared using a Leica UC7 ultramicrotome and observed using a transmission electron microscope (JEM1010; JEOL, Japan) after staining.

### Cuticular Wax Analysis

To analyze the cuticular wax, 4- to 5-week-old plants were used according to the method described in Zhou et al. (2014). Five of the second leaf blades (fresh weight about 1 g) from the top of wild type and *osabcg9-1* were immersed in 30 ml of preheated chloroform for 30 s at 60°C. Heneicosanoic acid and *n*-octacosane were added as internal standards. The extracted wax solvent was evaporated under nitrogen gas at 35°C. The dried wax mixtures were dissolved in 100 μl of pyridine and 100 μl of bis-*N,O*-(trimethylsilyl) trifluoroacetamide and derivatized by incubating them for 30 min at 90°C. The derivatized wax mixtures were re-dried under nitrogen gas and dissolved in chloroform. One microliter of wax samples was injected for GC–MS (GCMS-QP2010, Shimadzu, Tokyo, Japan; column 60-m HP-5, 0.32-mm i.d, df = 0.25 mm, Agilent Technologies, Palo Alto, CA, United States) and GC (GC-2010 Plus, Shimadzu, Tokyo, Japan; column 60-m DB-5, 0.32-mm i.d., df = 0.25 mm, Agilent Technologies, Palo Alto, CA, United States) for qualitative and quantitative composition analyses. We used the analysis conditions described in Lee and Suh (2015) with slight modifications. The sample was injected at 220°C, and the temperature was held for 4.5 min. The temperature was increased to 290°C at 3°C/min, maintained for 10 min, raised to 300°C at 2°C/min, and then maintained for 32 min at 300°C. The cuticular wax components were quantified from their peak area relative to the peak area of the internal standards. The area of the leaf blades was measured using Image J software (National Institutes of Health^2^).

### Cutin Polyester Analysis

Cutin analysis was performed as described in Li-Beisson et al. (2013). Cuticular wax extracted from leaf blades was immersed in 80°C isopropanol for 10 min. After cooling and grinding, samples were delipidated with chloroform:methanol (2:1 v/v), chloroform:methanol (1:2 v/v), and methanol solvent for at least overnight in each step. Delipidated samples were dried under nitrogen gas and re-dried in a vacuum desiccator for at least 24 h. Dry residues were depolymerized using the method of base catalysis. Briefly, 6 ml of reaction solvent and internal standards (methyl heptadecanoate and ω-pentadecalactone) were added to the dry residues. After heating for 2 h at 60°C, 10 ml of dichloromethane, 1 ml of glacial acetic acid, and 4 ml of 0.9% NaCl (w/v) were added. The lower organic phase, which is obtained after centrifugation, was washed with 2 ml of 0.9% NaCl (w/v) at least two times. Then the extracted solvent was treated with anhydrous sodium sulfate and dried under nitrogen gas. The transmethylated sample was acetylated with 100 μl of pyridine and 100 μl of acetic anhydride by heating at 60°C for 2 h. After evaporation under nitrogen gas, derivatized cutin monomers were dissolved in heptane:toluene (1:1, v:v) and injected for GC-MS and GC. The analysis protocol was as follows: injected at 110°C, raised by 2.5°C/min to 300°C, and held for 3 min at 300°C.

### Chlorophyll Leaching and Water Loss Analysis

The third leaves from the top at the tillering stage were collected and immersed in 30 ml of 80% ethanol. The chlorophyll concentration was quantified at 664 and 647 nm using a standard method from Lolle et al. (1997). The chlorophyll leaching amount was measured at 20, 40, 60, and 120 min as the percentage of total chlorophyll efflux at 24 h.

The rate of water loss was measured using the detached leaves at booting stage as described by Jenks et al. (1994). The second fully expand leaves were removed from plants and soaked in deionized water more than 2 h in dark. Then, the remaining water in the leaves was removed and dried in air. The leaves were weighed in low intensity light condition every 30 min for a total of 3 h. The rate of water loss was calculated by dividing weight at different time points dried in the air by the weight at the initial stage.

### Statistical Analysis

The data analysis was carried out with at least three biological replicates and the data was presented as the mean ± SE. Difference analysis was performed using Student’s *t*-test (two-tailed analysis) at *p*-value < 0.01 or *p*-value < 0.05.

## Result

### OsABCG9 Is Essential for Normal Development at the Vegetative Growth Stage

**FIGURE 1:**
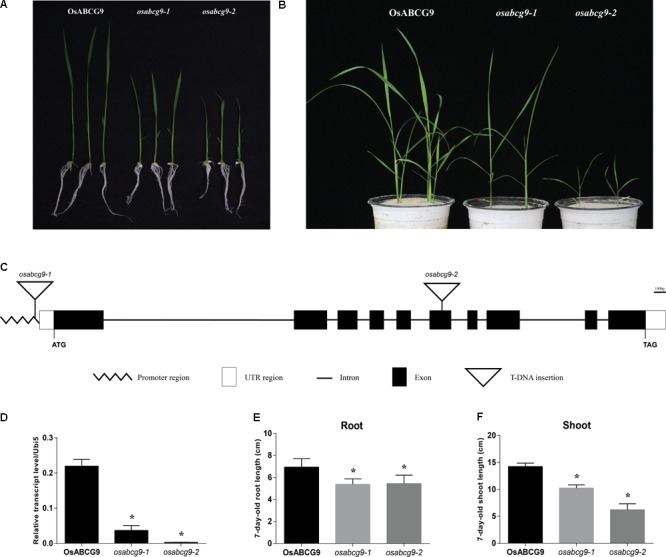
Phenotype analysis of *osabc9* mutants. Phenotypic comparison between wild type and mutant plants using **(A)** 1 week-old and **(B)** 4 week-old seedlings. **(C)** Schematic view of T-DNA insertions in ATG162 of *osabcg9-1* and the sixth exon of *osabcg9-2*. **(D)** Expression of *OsABCG9* in wild type plants and the two mutant alleles. Real-time PCR analysis was carried out with four independent biological replicates (asterisks indicate *p*-value < 0.01). *OsUbi5* gene was used as an internal control. Measurement of **(E)** root length and **(F)** shoot length between wild type plants and the two mutants was performed with ten independent biological replicates (asterisks indicate *p*-value < 0.05).

Among 16 leaf-preferred ABC transporter genes we identified before (Nguyen et al., 2014), we found two T-DNA insertional lines for the *OsABCG9* gene from the T-DNA insertional mutant pool (Jeon et al., 2000). Lines 1B-03134 (*osabcg9-1*) and 1B-22818 (*osabcg9-2*) interrupt the *OsABCG9* gene by T-DNA insertion (**Figure 1A**). Under field conditions, homozygotes of *osabcg9-1* exhibited a small phenotype (**Supplementary Figure S1**), but we failed to find any homozygous plants of the *osabcg9-2* mutant line. Therefore, those homozygous mutant plants were selected 7 days after germination on MSO media and transferred in soil to a greenhouse (**Figure 1B**). Under the greenhouse condition, the *osabcg9-2* homozygous mutant plants showed much more severe growth retardation than the *osabcg9-1* mutants and did not survive more than 1 month.

The predicted structure of *OsABCG9* comprises ten exons and nine introns. *osabcg9-1* has T-DNA located at the promoter region, about 162 bp upstream of the start codon, and the tagged T-DNA in *osabcg9-2* is at the sixth exon (**Figure 1C**). Quantitative RT-PCR analyses of *OsABCG9* expression in homozygous mutant plants indicated that the level of *OsABCG9* expression decreased by 10 times in *osabcg9-1* and by 100 times in *osabcg9-2*, compared with the wild type (**Figure 1D**). That result is consistent with the results at the phenotype level for two alleles caused by different T-DNA locations. At the 7-day-old seedling stage, the root length of the mutants was significantly shorter than that of the wild type; however, *osabcg9-1* and *osabcg9-2* did not differ from each other. In addition to reduced root size, the shoot height of *osabcg9-2* decreased by 57% compared with the wild type, and that of *osabcg9-1* decreased by 40% compared with the wild type (**Figures 1E, F**). These data indicate that OsABCG9 protein plays a crucial role in normal root and shoot development from the early seedling stage.

### OsABCG9 Alters Cuticular Permeability and Drought Sensitivity in Rice

**FIGURE 2:**
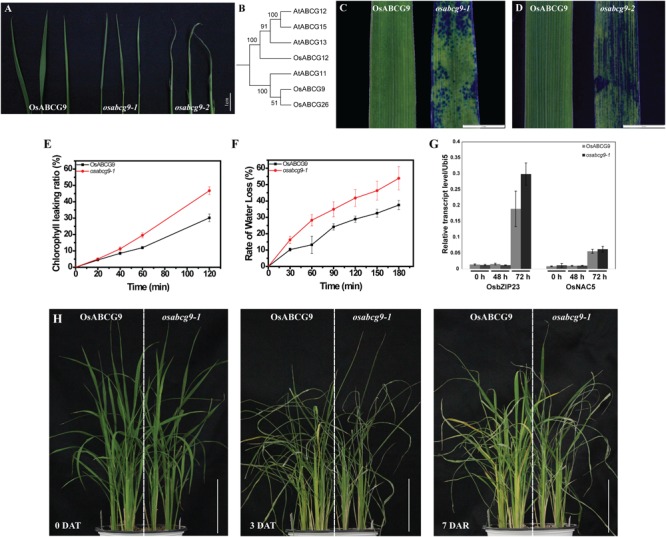
Altered cuticular permeability and drought sensitivity in wild type and *osabcg9-1*. **(A)** Comparison of water loss rate after exposure in air for up to 1 h between wild type and mutant plants. **(B)** Phylogenic analysis of 7 proteins similar to OsABCG9 in rice and *A. thaliana*. Staining result of 7-day-old leaves in 0.05% TBO solution: **(C)** *osabcg9-1* after 1 h and **(D)** *osabcg9-2* after 5 min. **(E)** Chlorophyll leaching assay showed that chlorophyll leaching occurred more quickly in *osabcg9-1* than in the wild type at every time point. The data was collected from three independent biological replicates. **(F)** Water loss rates of detached leaves of WT and *osabcg9-1*. The experiment was processed on six independent samples. **(G)** The expression of two drought-inducible marker genes, *OsbZIP23* and *OsNAC5*, at 0 h, 48 h, and 72 h. Each of expression level relative to internal control (*OsUbi5*) is the mean ± SE of three independent biological replications. **(H)** Plants were set up under water deficiency conditions for 3 days (DAT: days after treatment) and allowed to recover for 7 days (DAR: days after recovery). Bar 10 cm.

We observed that water loss in the two alleles is more severe than in the wild type, especially in *osabcg9-2*, after 1 h of exposure in the air (**Figure 2A**). Phylogenic analysis of seven genes with a high protein sequence similarity to *OsABCG9* in rice and *A. thaliana* revealed two clades of ABCG family members: *OsABCG9* was grouped with *OsABCG26* and *AtABCG11*, and *OsABCG12, AtABCG12, AtABCG15*, and *AtABCG13* belong to another group (**Figure 2B**). AtABCG11 protein is involved in wax and cutin transportation (Bird et al., 2007; Luo et al., 2007; Panikashvili et al., 2007). OsABCG26 protein is required for anther cuticle and pollen exine formation (Chang et al., 2016). Leaves of the wild type and the mutants were soaked in toluidine blue-o (TBO) solution. After 1 h, the *osabcg9-1* leaves had several spotted stains (**Figure 2C**), and *osabcg9*-2 got continuous stains after only 5 min, whereas the wild type was not stained (**Figure 2D**). These results suggest that OsABCG9 protein is likely important for cuticular wax deposition in rice.

As we mentioned above, water loss was much more severe in the mutants, *osabcg9-1* and *osabcg9-2*, than in the wild type. We also investigated whether cuticular permeability and sensitivity against water shortage were altered in the mutants. Subsequently, chlorophyll leaching, detached leaves and whole-plant drought-sensitivity assays were performed. The chlorophyll leaching rate is much higher in the *osabcg9-1* mutant than in the wild type (**Figure 2E**). After exposure to ethanol 80% for 2 h, 46.8% of the total chlorophyll was leached from *osabcg9-1* leaves, whereas the chlorophyll leaching from the wild type was 30.2%.

To evaluate the sensitivity of the mutation to drought condition, water loss rate from detached leaves were analyzed. At all the time points, *osabcg9-1* leaves lose water more rapidly than wild type leaves. After 3 h, loss water rate in detached leaves of *osabcg9-1* leaves was calculated as 54%, while the rate was 38% in detached leaves of wild type (**Figure 2F**).

To examine tolerance to drought stress at the plant level, wild type and mutant plants were germinated in the same pot. Water was withdrawn for 3 days and resupplied to recover stressed plants for 7 days. **Figure 2G** showed the expression of two drought-responsive marker genes including *OsbZIP23* (*LOC_Os02g52780*) and *OsNAC5* (*LOC_Os11g08210*) at 2 and 3 days after drought treatment (Xiang et al., 2008; Takasaki et al., 2010), informing that the applied drought stress well works. Under the water deficiency condition, the *osabcg9-1* plants recovered much more slowly than the wild type plants (**Figure 2H**). These results support that the mutation of *OsABCG9* alters cuticular permeability, making *osabcg9-1* more sensitive to drought stress than wild type plants.

### Anatomical Expression Patterns of *OsABCG9* Using RT-PCR and Histochemical GUS Analyses

**FIGURE 3:**
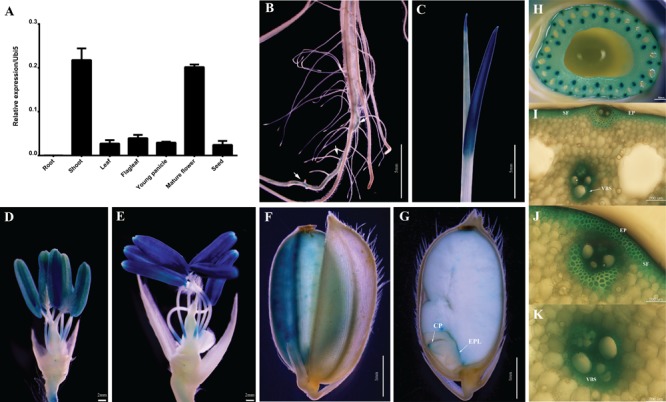
Expression profiles of *OsABCG9* in anatomical tissues using GUS and RT-PCR analyses. **(A)** Anatomical expression from root, shoot, leaf, flag leaf, young panicle, mature flower, and seed. The data is presented the mean of four independent biological replicates with ± SE. *OsUbi5* gene was used as an internal control. Histochemical staining of GUS activity in *pOsABCG9::GUS* transgenic plants: **(B)** root, **(C)** young leaf, **(D,E)** anther, **(F)** seed coat, **(G)** coleoptile, **(H)** stem, **(I,J)** epidermal cells, **(I,J)** sclerenchymatous fiber tissue, **(I,K)** vascular bundler sheath. Arrow indicates GUS expression in root. EP: epidermal cell, VBS: vascular bundler sheath, SF: sclerenchymatous fiber, CP: coleoptile, EPL: epithelium layer.

The expression level of *OsABCG9* was determined in various organs/tissues (root, shoot, leaf, flag leaf, young panicle, mature panicle, and seed) by quantitative RT–PCR using a pair of forward primers at the eighth exon and a reverse primer at the nineth exon (**Supplementary Table S1**). As shown in **Figure 3A**, the expression of *OsABCG9* was highest in the shoots at the vegetative stage and the mature flower at the reproductive stage (**Figure 3A**). The tissue-specific expression was analyzed with *OsABCG9* promoter-GUS transgenic plants. GUS activity was detected weakly in the seminal root (**Figure 3B**) and strongly in the young shoot (**Figure 3C**), anther (**Figures 3D,E**), and especially in the mature anther and stem (**Figure 3H**). Interestingly, the reporter gene was also expressed in epidermal cells, sclerenchymatous fiber tissue in the stem (**Figures 3I,J**), and the vascular bundler sheath (**Figure 3K**). GUS expression also occurred in the seed coat (**Figure 3F**), coleoptile and epithelium layer of the embryo (**Figure 3G**).

### Subcellular Localization Analysis Inform the Role of OsABCG9 in Plasma Membrane

**FIGURE 4:**
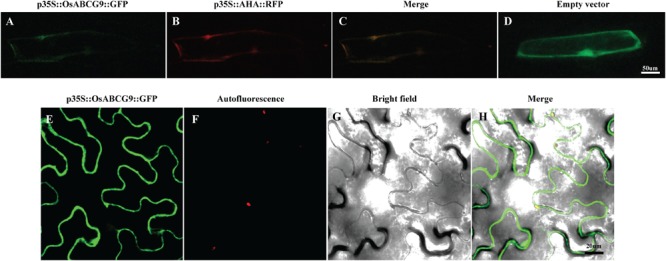
Sub-cellular localization of OsABCG9::GFP in onion skin epidermal cells and tobacco epidermal cells. Onion skin epidermal cells expressing OsABCG9::GFP **(A)**, AHA::RFP **(B)**, and both **(C)**, showing fluorescent signals in the plasma membrane, whereas GFP alone was expressed in the cytosol, nucleus, and plasma membrane **(D)**. Fluorescence image of OsABCG9::GFP fusion protein on tobacco leaves **(E)**, auto-florescence **(F)**, and bright field image of OsABCG9::GFP **(G)** and overlay of three channels **(H)**.

A protein localization tool^3^ predicted that OsABCG9 protein would be located in the plasma membrane. To investigate the subcellular localization of OsABCG9 protein, we bombarded onion epidermal cells with a solely negative control (empty vector), *p35S::GFP* construct, co-bombarded them with a *p35S::OsABCG9-GFP* fusion construct and a positive control construct characterized for plasma membrane localization (*p35S::AHA2–RFP* fusion vector). After 24 h of incubation in darkness, the onion cells transformed by the *p35S::GFP* construct showed fluorescence throughout the cells, including the cytosol, nucleus, and plasma membrane (**Figure 4D**). However, the GFP signal from the OsABCG9-GFP (**Figure 4A**) overlapped with the RFP signal (**Figure 4B**) driven by the AHA2 protein (**Figure 4C**), demonstrating that OsABCG9 protein is localized at the plasma membrane. In addition, transient expression of the OsABCG9-GFP fusion protein in tobacco was carried out. GFP signal in OsABCG9-GFP (**Figure 4E**) well overlapped with plasma membrane of tobacco leaf (**Figure 4H**), while **Figures 4F and 4G** showed auto-florescence and bright field image, respectively, further supporting the data in onion cells.

### OsABCG9 Affects Cuticular Wax Accumulation and Composition in Rice

**FIGURE 5:**
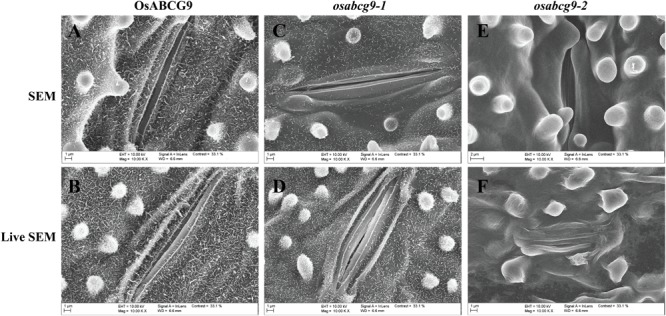
Scanning electron microscope (SEM) analysis of wild type and mutant plants. Epicuticular wax crystal density in **(A,B)** wild type plants is much greater than in **(C,D)** *osabcg9-1* mutant. Epicuticular wax crystals vanished entirely from *osabcg9-2* mutant **(E,F)**. SEM indicates treated samples **(A,C,E)**, and LIVE SEM indicates untreated samples **(B,D,F)**.

To address whether the OsABCG9 protein is involved in the composition of the cuticular lipid, we observed the detailed leaf surface of wild type, *osabcg9-1*, and *osabcg9-2* samples by scanning electron microscopy (SEM). To more accurately determine differences in the leaf surfaces between the wild type and transgenic plants, we also observed wax crystals on the leaf surfaces of intact samples. In the wild type plants, platelet-type wax crystals densely covered the leaf surface (**Figures 5A,B**). Unlike the wild type plants, fewer and smaller wax crystals were observed on the surfaces (including the papillae) of the *osabcg9-1* plants (**Figures 5C,D**). Notably, wax crystals vanished completely from the leaf surface of the *osabcg9-2* samples (**Figures 5E,F**). These results suggest that a loss of function of *OsABCG9* reduced cuticular wax accumulation on rice leaves in the mutant plants.

**FIGURE 6:**
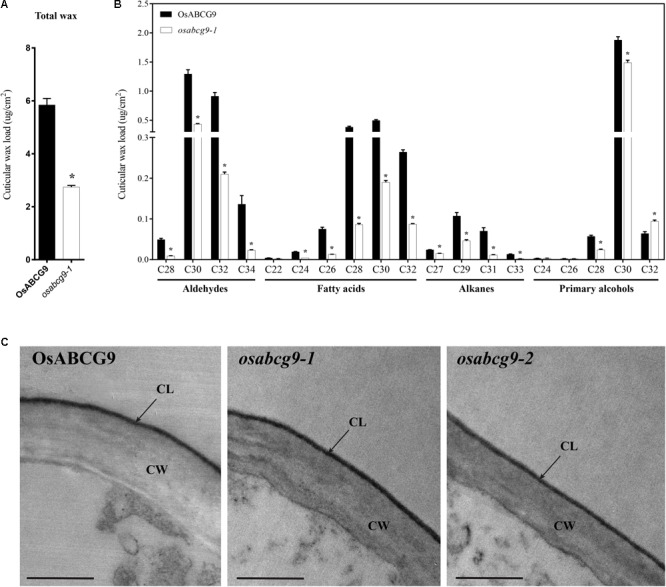
Cuticular wax composition and amount in wild type and *osabcg9-1* leaves by GC-MS/ GC and TEM analyses of wild type and mutant samples. Comparison of the total wax load **(A)** and individual wax components **(B)** in wild type and *osabcg9-1* plants. Each value is the mean ± SE of seven independent measurements. Asterisks indicate *p*-value < 0.01 using a student’s *t*-test. **(C)** TEM analyses of wild type and mutant samples. CL: cuticular layer, CW: cell wall. Bar 0.5 μm.

Because the *osabcg9-2* homozygous mutant plants showed much more severe growth defects during the vegetative growth stage, we used gas chromatography–mass spectrometry (GC–MS) and GC analyses to compare the cuticular wax components and amounts between only the wild type and *osabcg9-1* to gain more knowledge about the function of *OsABCG9* gene. The total amounts of cuticular wax decreased dramatically in *osabcg9-1* leaves, equivalent to 53% of that in wild type leaves (**Figure 6A** and **Supplementary Table S2**). This analysis showed that the alterations in *osabcg9-1* caused the loss of individual cuticular wax components: (1) aldehydes, (2) fatty acids, and (3) alkanes (**Supplementary Table S2**). Most of the individual aldehydes with a carbon chain length C28–C34, fatty acid chain length C24–C32, alkane chain length C27–C33, and primary alcohols with chains length C28–C30 were reduced in this mutant, compared with the wild type (**Figure 6B**). In contrast, a compound of primary alcohol with a carbon chain length of C32 in *osabcg9-1* increased 46% compared to the wild type, but that did not seem to create any significant change because of the small amount of the C32 compound (**Supplementary Table S1**). Thus, the overall level of primary alcohol compounds also decreased. The loss of function analysis for *OsABCG9* therefore resulted in altered levels and composition of the wax in the cuticle.

The reduction of cuticular wax led us to examine whether the *osabcg9-1* lines had altered cutin monomers in the leaves compared to the wild type. An ultrastructural analysis of the leaf cuticle by transmission electron microscopy (TEM) on wild type and mutant samples indicated that all of them showed even thickness, regular compactness, and a clear cuticle boundary (**Figure 6C**). GC-MS and GC analysis of the cutin monomer components between the wild type and *osabcg9-1* mutant also showed no significant difference in their amounts of cutin monomers (**Supplementary Figure S2**).

## Discussion

We have shown that *OsABCG9* gene is involved in wax accumulation in rice by analyzing two independent T-DNA insertional mutants. These *osabcg9* mutants exhibited a drought sensitive phenotype. Phylogenetic analysis of ABCG family members close to OsABCG9 suggests that OsABCG9 plays roles in cuticular composition in epidermal tissues. In addition, microscope and cuticular component analyses verified that only wax, not cutin, was reduced in the *osabcg9* mutants.

Many genes already identified as enzymes that catalyze the formation of wax are localized in the ER of epidermal cells (Kunst and Samuels, 2003; Zheng et al., 2005; Greer et al., 2007; Kim S. et al., 2013). Wax molecules are delivered to the plasma membrane by an unknown pathway and then released to the apoplast by a transporter. It is supposed that wax transporters are localized at the plasma membrane of epidermal cells. GUS expression under a native promoter of *OsABCG9* was detected below the epidermal cells in sub-epidermal cells (sclerenchymatous fiber tissue), roots, seed coats, and vascular bundler sheath cells. This expression pattern overlaps highly with that of *AtABCG11* (Bird et al., 2007; Luo et al., 2007; Panikashvili et al., 2007). In addition to histochemical GUS experimentation, previous studies attempted to prove the precise expression of *AtABCG11* by using the construct *Pro_AtABCG11_::GFP-AtABCG11*. GFP signals displayed a restricted epidermal distribution pattern in the stem. Thus, Luo et al. (2007) supposed that GFP signals did not occur in sub-epidermal cells. In fact, studies using a tag such as GFP at the N-terminal of AtABCG11, which does little to preserve the localization of the native protein (Palmer and Freeman, 2004), might not precisely demonstrate the expression of *AtABCG11* in epidermal or sub-epidermal cells or both. Therefore, *OsABCG9* (as well as AtABCG11) promoter-reporter expression might not be confined to the epidermal layer, suggesting that it might participate in the transportation of other substrates, such as lipid-derived compounds. For example, some reports have shown that genes with expression in the roots and seed coat are also associated with wax metabolism: *KCS1* (Todd et al., 1999), *CER5* (Pighin et al., 2004), *AtABCG11* (Panikashvili et al., 2007), and *KCS9* (Kim J. et al., 2013). Those genes are involved in transporting suberin, another lipid-derived chemical that is similar in structure to the cuticular lipids. Because *OsABCG9* expression goes beyond epidermal cells to the detection of GUS expression in roots, seed coats, vascular bundles, sclerenchymatous fiber tissue in the stem and epithelium layer of the embryo, we suppose that OsABCG9 protein might transport more lipid-like molecules in addition to wax compounds. Further research is needed to understand the complete function of OsABCG9 protein.

Various land plants show diversity in the shape, size, and arrangement of wax crystals, based on the composition, biogenesis, and function of those surface structures. Wax crystals in rice are shaped like platelets, whereas those in *Arabidopsis* are longitudinal bundles of rodlets. Platelets are formed primarily of aldehydes, ketones, or primary alcohols (Macey and Barber, 1970; Rhee et al., 1998; Riedel et al., 2003), and rodlets are formed primarily from alkanes and alcohols (Jenks et al., 1995; Rashotte et al., 2001). Although OsABCG9 is estimated to have 78% similarity with the *Arabidopsis* protein AtABCG11, there are obvious differences between them in function. *AtABCG11* mutants showed decreased wax accumulation because of a large reduction in the alkane nonacosane (C29) (Bird et al., 2007; Luo et al., 2007; Panikashvili et al., 2007). Unlike *Arabidopsis*, rice wax is strengthened by aldehydes (Haas et al., 2001), and the component of wax most reduced in the *OsABCG9* mutants was aldehydes, especially triacontanal (C30) and dotriacontanal (C32) compounds. Thus, OsABCG9 protein plays important roles in maintaining the stability of the epidermal wax layer in rice. Furthermore, *osabcg9-1* lost 53% of its wax, and wax crystals disappeared completely in *osabcg9-2* (by SEM analysis), whereas a load loss of 40–60% of wax was observed in the weakest *atabcg11* mutant (Bird et al., 2007; Luo et al., 2007; Panikashvili et al., 2007). In addition, biochemical analysis revealed that OsABCG9 might transport almost wax compounds, even though AtABCG11 transported only a few. In other words, OsABCG9 in rice might have roles in wax secretion unlike those of AtABCG11 in *Arabidopsis*.

In *Arabidopsis*, AtABCG11 forms functional complexes, such as homodimers or heterodimers with AtABCG12. In contrast, the activity of the AtABCG12 protein depends on its interaction with AtABCG11 (McFarlane et al., 2010). AtABCG11 is involved in both cutin and wax transportation (Bird et al., 2007; Luo et al., 2007; Panikashvili et al., 2007), whereas AtABCG12 is involved solely in wax transportation (Pighin et al., 2004). Thus, AtABCG12 retains an overlapped function with AtABCG11 in wax secretion. Interestingly, we concluded that OsABCG9, the ortholog of AtABCG11 in rice, plays a unique function in transporting wax but not cutin. Other ABCG genes might be responsible for transporting cutin on the leaf surface. Further studies will need to clarify the function of those as-yet uncharacterized ABCG genes in rice that might form heterodimers with OsABCG9.

## Author Contributions

K-HJ, GA, and MCS conceived and designed the research plans. VN performed most of the experiments. SBL carried out the cuticular wax analysis. K-HJ and VN designed the experiments and analyzed the data. K-HJ, MCS, SBL, and VN wrote the paper.

## Conflict of Interest Statement

The authors declare that the research was conducted in the absence of any commercial or financial relationships that could be construed as a potential conflict of interest.

## Footnotes

1 http://signal.salk.edu/cgi-bin/RiceGE

2 http://rsb.info.nih.gov/ij

3 http://www.softberry.com

## Supplementary Material

FIGURE S1 Comparison of the phenotype of wild type plant and *osabcg9-1* plants in booting stage.

FIGURE S2 Amounts of cutin monomers in wild type and *osabcg9-1* leaves by GC-MS. The total cutin load **(A)** and total of each component of cutin **(B)** between the wild type and *osabcg9-1* samples were similar. Each value is the mean ± SE of four independent measurements.

TABLE S1 Sequences of the primers used for genotyping, real-time PCR amplification, histochemical GUS assay, and subcellular localization.

TABLE S2 Details of individual wax composition analysis by GC-MS of wild type and *osabcg9-1* samples.
