# Supplementary figures and images for: OsABCG9 Is an Important ABC Transporter of Cuticular Wax Deposition in Rice
Source: Front Plant Sci. 2018 Aug 7;9:960. doi: 10.3389/fpls.2018.00960 (PMC6091143; doi:10.3389/fpls.2018.00960)

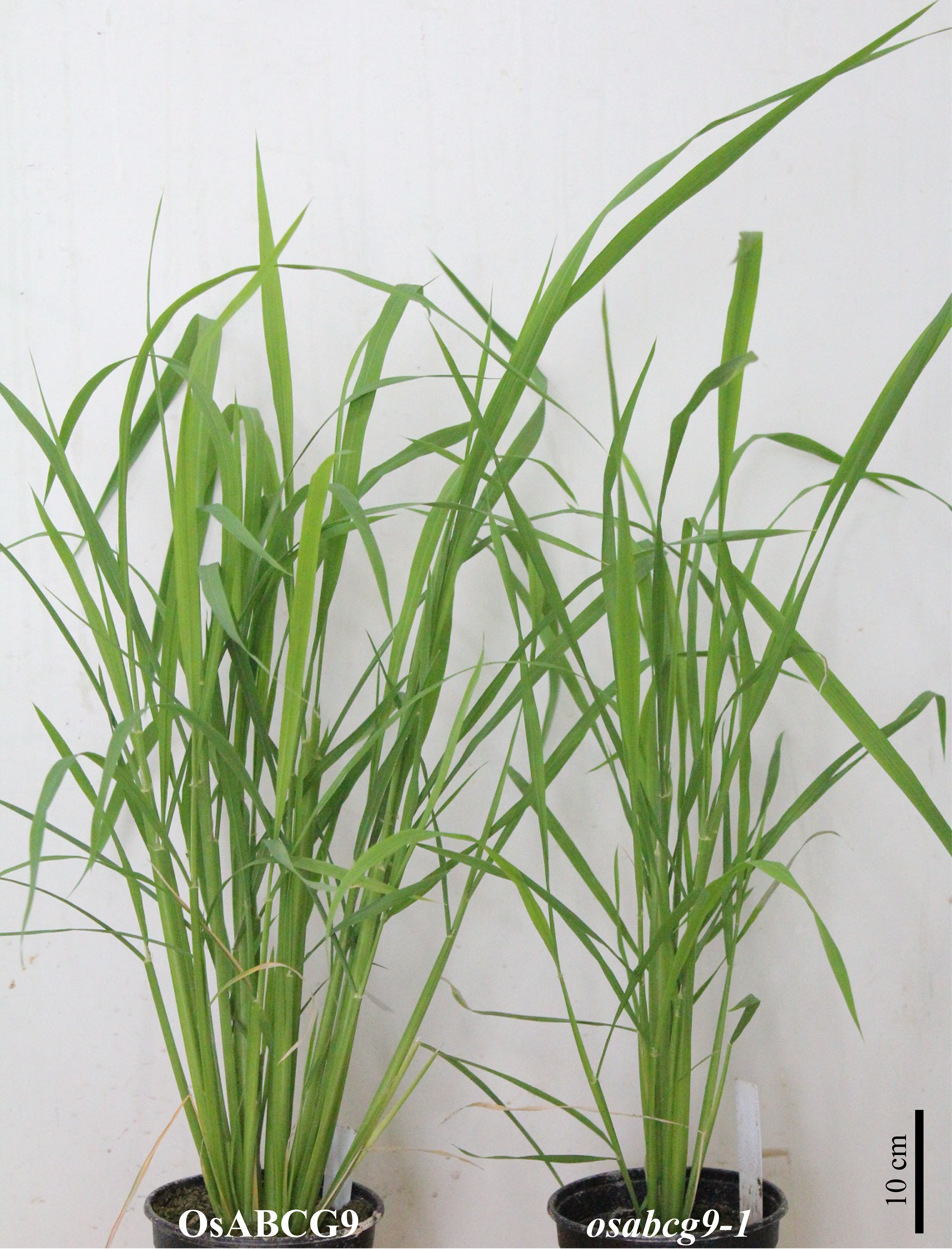

Supplement: FIGURE S1 — Comparison of the phenotype of wild type plant and osabcg9-1 plants in booting stage. [file Image_1.TIF]

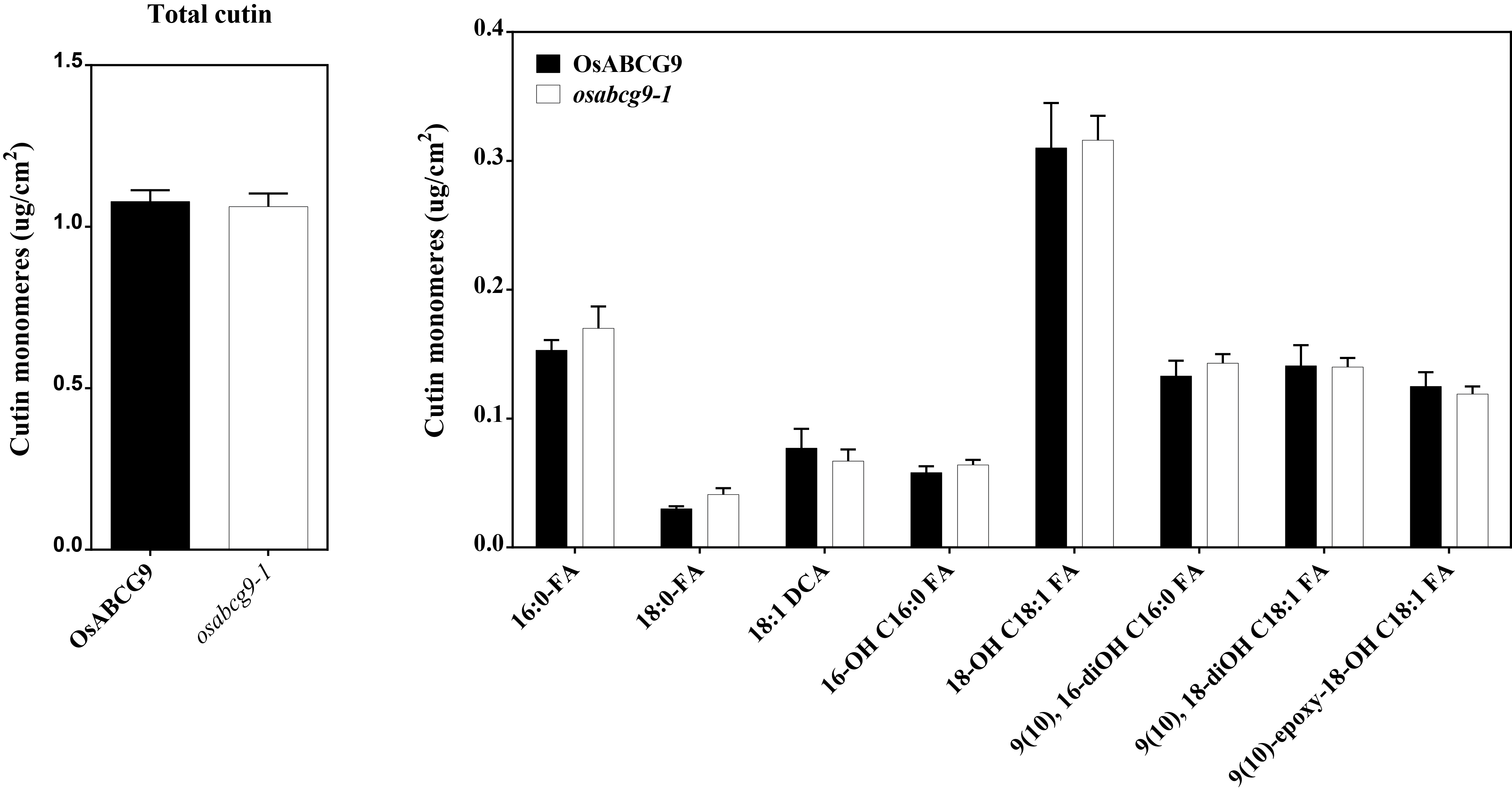

Supplement: FIGURE S2 — Amounts of cutin monomers in wild type and osabcg9-1 leaves by GC-MS. The total cutin load (A) and total of each component of cutin (B) between the wild type and osabcg9-1 samples were similar. Each value is the mean ± SE of four independent measurements. [file Image_2.TIF]
