# Supplementary material for: OsABCG9 Is an Important ABC Transporter of Cuticular Wax Deposition in Rice
Source: Front Plant Sci. 2018 Aug 7;9:960. doi: 10.3389/fpls.2018.00960 (PMC6091143; doi:10.3389/fpls.2018.00960)
Supplement: TABLE S2 — Details of individual wax composition analysis by GC-MS of wild type and osabcg9-1 samples. [file Table_2.DOCX]

|  |  | OsABCG9 | *osabcg9-1* | Reduction of wax content (%) |
| --- | --- | --- | --- | --- |
| Fatty acid | C22 | 0.004 ± 0.001 | 0.002 ± 0.001 | 50 |
|  | C24 | 0.019 ± 0.003 | 0.004 ± 0 | 78.94 |
|  | C26 | 0.075 ± 0.012 | 0.013 ± 0.001 | 82.66 |
|  | C28 | 0.381 ± 0.045 | 0.086 ± 0.008 | 77.42 |
|  | C30 | 0.495 ± 0.043 | 0.19 ± 0.012 | 61.61 |
|  | C32 | 0.264 ± 0.015 | 0.087 ± 0.004 | 67.04 |
| Aldehydes | C28 | 0.049 ± 0.008 | 0.009 ± 0.001 | 81.63 |
|  | C30 | 1.293 ± 0.196 | 0.432 ± 0.026 | 66.58 |
|  | C32 | 0.911 ± 0.175 | 0.21 ± 0.014 | 76.94 |
|  | C34 | 0.136 ± 0.057 | 0.023 ± 0.003 | 83.08 |
| Primary alcohols | C24 | 0.003 ± 0.001 | 0.003 ± 0.002 | 0 |
|  | C26 | 0.002 ± 0.001 | 0.002 ± 0 | 0 |
|  | C28 | 0.057 ± 0.007 | 0.024 ± 0.005 | 57.89 |
|  | C30 | 1.877 ± 0.151 | 1.488 ± 0.116 | 20.72 |
|  | C32 | 0.064 ± 0.012 | 0.094 ± 0.008 | -46.87 |
| Alkanes | C27 | 0.024 ± 0.001 | 0.015 ± 0.001 | 37.5 |
|  | C29 | 0.107 ± 0.023 | 0.046 ± 0.007 | 57.00 |
|  | C31 | 0.07 ± 0.022 | 0.011 ± 0.003 | 84.28 |
|  | C33 | 0.013 ± 0.002 | 0.002 ± 0 | 84.61 |
| Total |  | 5.845 ± 0.648 | 2.741 ± 0.164 | 53.10 |

Table S2: Detail of individual wax composition amount analysis by GC-MS of wildtype and *osabcg9-1*
